# A new species of the genus *Proutia* Tutt (Lepidoptera, Psychidae) from Korea, based on morphology and DNA barcodes

**DOI:** 10.3897/BDJ.11.e110313

**Published:** 2023-10-05

**Authors:** Dong-June Lee, Jae-Seok Lee, Jongwon Kim, Hyeon Lee, Bong-Kyu Byun, Seung Jin Roh

**Affiliations:** 1 Honam National Institute of Biological Research, Mokpo, Republic of Korea Honam National Institute of Biological Research Mokpo Republic of Korea; 2 Hannam University, Daejeon, Republic of Korea Hannam University Daejeon Republic of Korea

**Keywords:** *Proutiacornucervae*, *Bruandellaniphonica*, new combination, DNA barcode, bagworms, Korea

## Abstract

**Background:**

The genus *Proutia* Tutt, 1899 (Lepidoptera, Psychidae) comprises 14 species found throughout the world. In East Asia, three species, *Proutiachinensis* Hättenschwiler & Chao, 1990, *P.maculatella* Saigusa & Sugimoto, 2014 and *P.nigra* Saigusa & Sugimoto, 2014, are known from Korea, Japan and China.

**New information:**

*Proutiacornucervae* Roh & Lee, sp. nov. is newly recognised from Korea. In addition, *Bruandellaniphonica* (Hori) is transferred to genus *Proutia*. Male and genitalia of the species are described and DNA barcodes are provided.

## Introduction

The family Psychidae consists of 241 genera with 1,350 described species (van Nieukerken et al. 2011). Phylogenetically, Psychidae have been placed in the superfamily Tineoidea (Regier et al. 2015). The larvae of the family Psychidae usually make cases in unique shapes for each species (Sugimoto 2009a, Sugimoto 2009b).

The genus *Proutia* Tutt, 1899 was based on the type species *Psyche betulina* Zeller, 1839. Recently, genera *Anaproutia* Lewin, 1949 and *Bruandella* Saigusa & Sugimoto, 2014 were synonymised to genus *Proutia* (Arnscheid and Weidlich 2017). In total, 14 species of the genus *Proutia* are known worldwide, with all species being distributed throughout the Palaearctic Regions (Sobczyk 2011, Saigusa and Sugimoto 2014, Arnscheid and Weidlich 2017). In East Asia, three species, *Proutiachinensis* Hättenschwiler & Chao 1990, *P.maculatella* Saigusa & Sugimoto, 2014 and *P.nigra* Saigusa & Sugimoto 2014, are known from Korea, Japan and China (Sobczyk 2011, Saigusa and Sugimoto 2014, Roh et al. 2016, Roh and Byun 2017). The genus *Proutia* is known, based on the following adult characters: labial palp reduced to one segment and antenna bipectinate; nine veins arising from the discal cell, intercalary cell present in the forewing; genitalia of the male usually with short anellus and saccus, vesica without cornuti. The larvae build their cases by putting together debris of algae, bark and wood (Arnscheid and Weidlich 2017).

The purpose of this paper is to describe a new species, *Proutiacornucervae* sp. nov., including the collecting localities, illustrations of male adult and genitalia and DNA barcode. In addition, *Bruandellaniphonica* (Hori, 1926) is transferred to genus *Proutia*. Furthermore, DNA barcodes for precise identification of five species of Korean *Proutia* (four) and *Psyche* (one) are also provided.

## Materials and methods

The materials examined in this study are kept in the Entomological Collection, Honam National Institute of Biological Resources (ECHNIBR), Mokpo, Korea. The male genitalia were dissected and examined after mounting on glass slides in 80% glycerol solution. The wing venations were examined in 70% alcohol solution. Photographs of adults were taken using a MP-E 65 mm f/2.8 1-5x Macro Photo, attached to 5D Mark IV digital camera (Canon, Tokyo, Japan). Photographs of the male genitalia was taken using a DFC 95 mm digital camera (Leica, Wetzlar, Germany) attached to a Leica M205A stereomicroscope (Leica, Wetzlar, Germany). Terminology and morphological characters of the adult, wing venation and genitalia follow Saigusa and Sugimoto (2014) and Arnscheid and Weidlich (2017).

Genomic DNA from seven specimens of *Proutiamaculatella*, four specimens of *P.nigra*, nine specimens of *P.niphonica*, one specimen *P.cornucervae* sp. nov. and one specimen of *Psyche yeongwolensis* was extracted from the legs of dried specimens of adults in 100% alcohol using a Genomic Cell/Tissue Spin Mini Kit (Qiagen, Inc, Hilden, Germany), according to the manufacturer’s protocol. Specimens were sequenced and the DNA barcode, cytochrome oxidase subunit I gene (*COI*), was amplified using the primers LCO1490 and HCO2198 (Folmer et al. 1994). Polymerase chain reaction (PCR) conditions for amplification followed the manufacturer’s protocol (Platinum Taq, Invitrogen, Carlsbad City, CA, USA). The amplicons were purified using the QIAquick® PCR purification kit (QIAGEN, Inc, Hilden, Germany) and directly sequenced at Macrogen (Seoul, Korea). Contigs were assembled in Geneious prime (Kearse et al. 2012). Successful sequences were uploaded to GenBank (Table 1).

The barcodes were compared to 50 DNA barcodes of the genera *Proutia* and *Psyche* downloaded from NCBI (https://www.ncbi.nlm.nih.gov/) and BOLD systems (https://v4.boldsystems.org/) (Table 1). A Neighbour-Joining analysis (NJ) was performed with MEGA X (Kumar et al. 2018) using the Kimura-2-Parameter (K2P) model (Kimura 1980) for nucleotide substitutions. Bootstrap support values for each node were also evaluated via MEGA X with 1000 replicates. Parsimony (PA) with bootstrap analyses were conducted in TNT 1.5 (Goloboff and Catalano 2016).

## Taxon treatments

### Proutia cornucervae

Roh & Lee sp. nov.

085388DF-866F-5FC2-BDBB-5811E6888E32

B3404205-CD97-4E82-87A6-2B539EEF6876

#### Materials

- **Type status:** Holotype. **Occurrence:** recordNumber: MT154331; individualID: MT154331; individualCount: 1; otherCatalogNumbers: GBMND76673-21; occurrenceID: DF1C4BA7-55A1-598A-96D7-AA9131C8DC18; **Taxon:** scientificName: *Proutiacornucervae* Roh & Lee, sp. nov.; phylum: Arthropoda; class: Insecta; order: Lepidoptera; family: Psychidae; **Location:** country: South Korea; stateProvince: Daejeon-si; county: Yuseong-gu; decimalLatitude: 36.3333; decimalLongitude: 17.3333; **Event:** year: 2015; month: 4; day: 12; **Record Level:** institutionCode: Mined from GenBank, NCBI

#### Description

**Figure 1.**
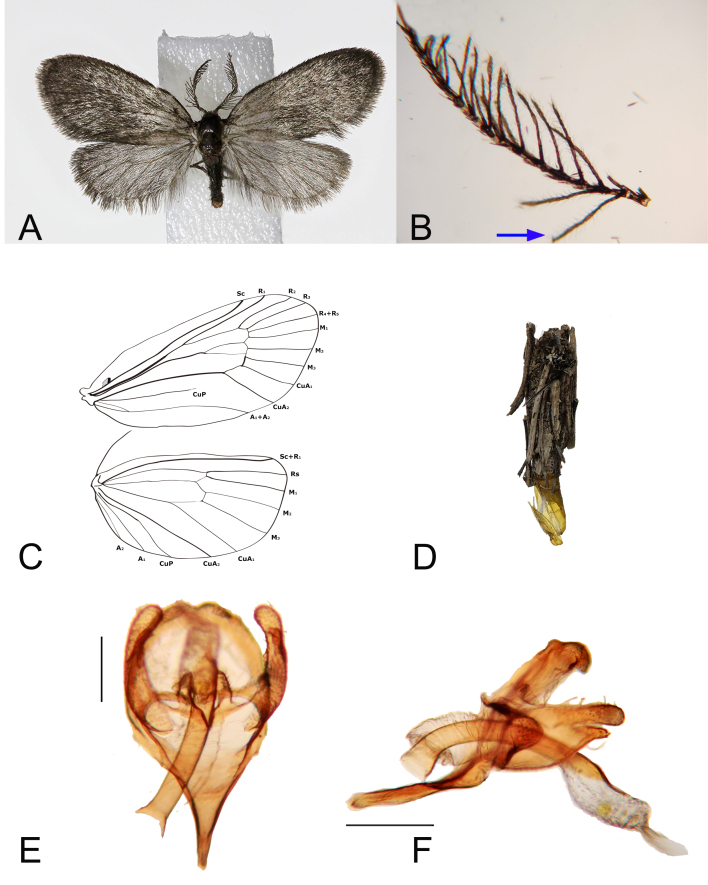
Male of *P.cornucervae* sp. nov., Holotype: **A** adult; **B** antenna; **C** wing venation; **D** larval case; **E** genitalia, dorso-ventral aspect; **F** ditto, lateral aspect.

**Adult** (Fig. 1A-C, E and F). **Male. Head**: Vertex of head densely clothed with brown hairs; ocelli absent; antennae less than 1/3 length of forewing, flagellum bipectinated. **Thorax**: notum covered with dark-brown scales. Wingspan 14 mm. Forewing dark-brown scale covered, generally without markings on upperside; 6.8 mm in length excluding fringe with termen distinctly formed; median cell 0.69 times as long as forewing; accessory cell absent; intercalary cell present; Sc and R_1_ terminating at 4/5 costa; R_2_ and R_3_ stalked at anterior part of the cell; R_4_ and R_5_ originating at corner of anterior part of cell to reach apex; M_2_ stalked at corner of intercalary cell; CuA_1_ and CuA_2_ parallel to tornus. Hindwing covered with greyish scales; 4.9 mm excluding fringe, with termen distinctly formed and gently curved; median cell 0.59 times as long as hindwing; Rs and M_1_ separated. Legs covered with dark brown scales. **Abdomen**: Male genitalia with tegumen wide, rounded; uncus formed as rectangular; saccus straight, slightly long and slender; apical part of ampulla gently arched with club shape, setae present, length of ampulla 0.45 times as long as length of valva dorsal margin; phallus curved, cornuti wide and long. In dorso-ventral aspect, uncus concave; gnathos absent; valva slightly narrow, apical part of valva presented with short setae; apical margin of harpe formed into three weakly-rounded laciniation; juxta absent; anellus well developed, pointed, 0.3 times as long as length of valva; phallus long. **Larval case** (Fig. 1D). 9 mm in length. Larvae attach their tiny and slender branches on to the larval case of cylindrical shape.

#### Diagnosis

This species is similar to *P.nigra* Saigusa and Sugimoto, but can be distinguished by the much darker wings being blackish-brown and pectinations of the antennae elongate and about about same length from basal flagellomeres to flagellomere 9. Whereas in *P.nigra*, the wings are slightly lighter, being brownish-black and pectinations of antennae abruptly becoming longer to 7^th^ flagellomere.

#### Etymology

The specific name is derived from the Latin cornu and cerva (= antler), referring to the antennae shape.

#### Distribution

Korea (new species).

### Proutia niphonica

(Hori, 1926), comb. nov.

A1874E64-F6DF-5741-BD88-44196A8626DC

Eumea niphonica Hori, 1926: 28 (*Eumea* is misspelling of *Fumea*). Type locality: Japan.
Psyche casta (Pallas, 1767): Sauter and Hättenschwiler (1991): 79; Leraut (1997): 87; Sobczyk (2011): 257.
Bruandia niphonica (Hori, 1926): Sugimoto (2009a): 12; Saigusa and Sugimoto (2013): 145.
Bruandella niphonica (Hori, 1926): Saigusa and Sugimoto (2014): 143; Roh and Byun (2017): 224; Saigusa and Sugimoto (2022): 147.

#### Notes

**Figure 2.**
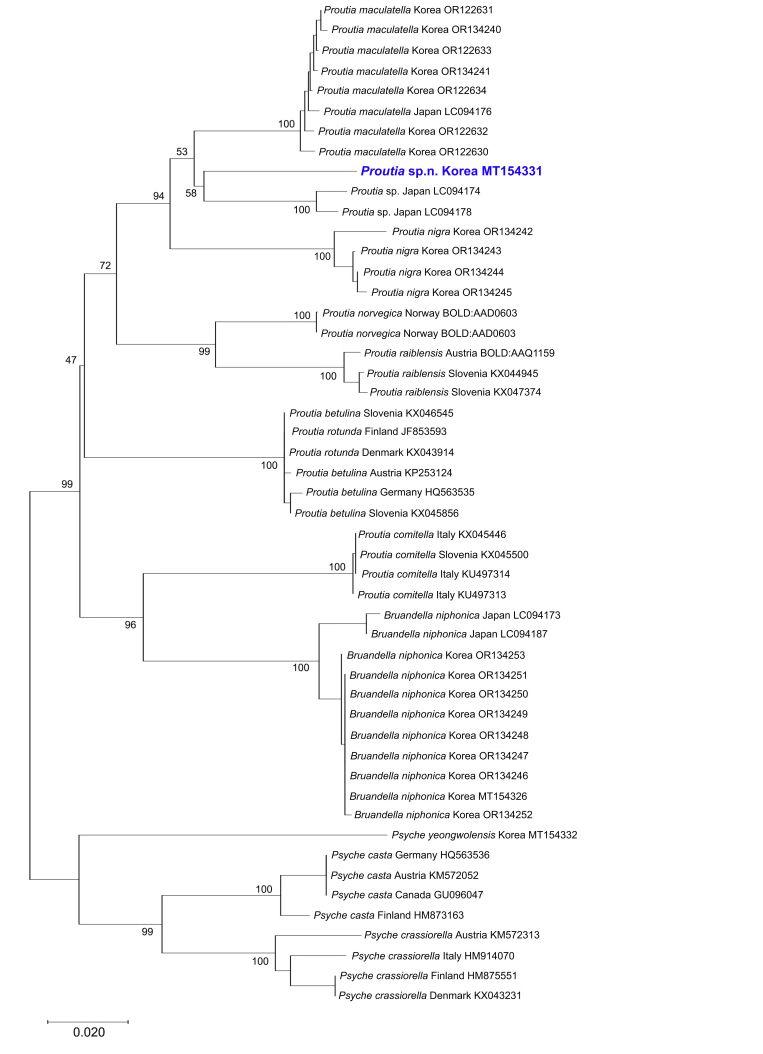
Neighbour-Joining tree, based on partial *COI* gene sequences with bootstrap values. Scale bar indicates the expected number of substitutions per site.

**Figure 3.**
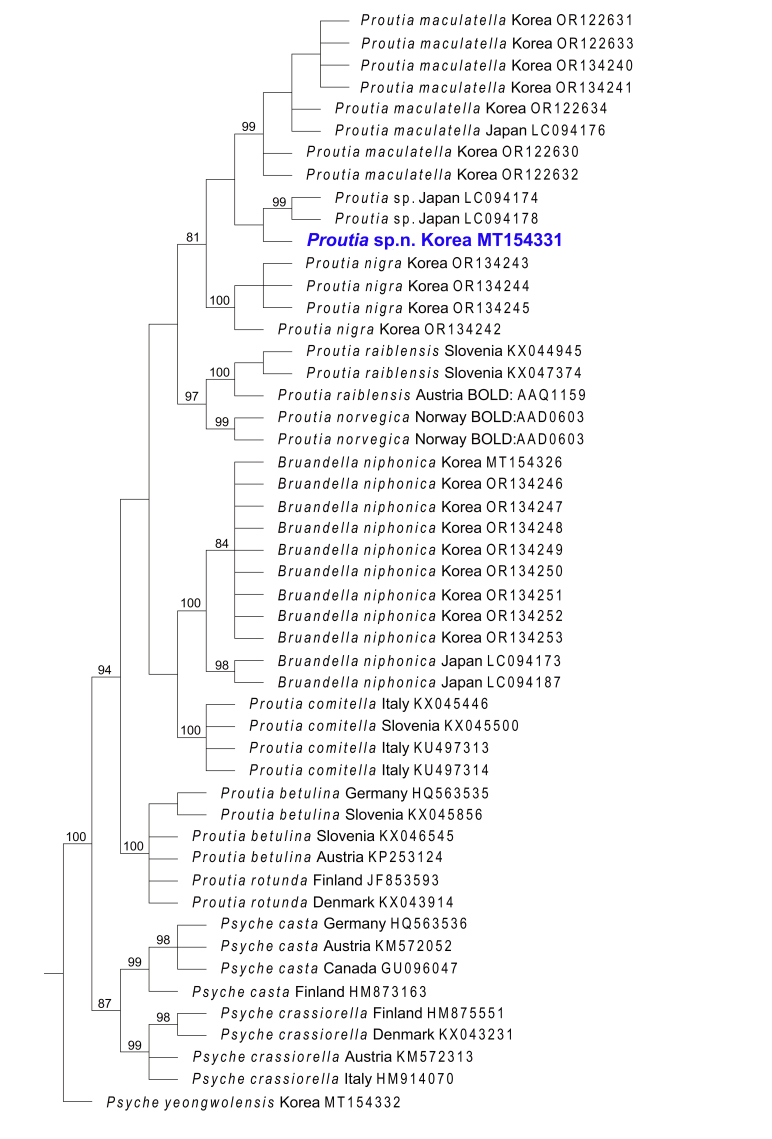
Strict consensus tree of equally parsimonious cladograms, based on partial *COI* gene sequences with bootstrap values (bootstrap values over 80% are indicated).

The placement of *niphonica* Hori, 1926 has been a debatable issue. The species was described by Hori, based on specimens collected from Honshu and Kiyshu in Japan and assigned to *Eumea*, an incorrect subsequent spelling of *Fumea* Haworth, 1812. Sauter and Hättenschwiler (1991) treated it as a junior subjective synonym of *Psychecasta* (Pallas, 1767) and this treatment has been subsequently followed by Leraut (1997), Sobczyk (2011) and Arnscheid and Weidlich (2017). However, Sugimoto (2009a) treated it as valid species in *Bruandia* Tutt, 1900 and this was followed by Saigusa and Sugimoto (2013). *Bruandia* Tutt is a homonym of *Bruandia* Desmarest, 1857. Sauter and Hättenschwiler (1999) proposed *Anaproutia* Lewin, 1949 as a replacement name of *Bruandia* Tutt and then *Anaproutia* was treated as a synonym of *Proutia* by Bengtsson and Palmqvist (2008). Saigusa and Sugimoto (2014) erected *Bruandella* as a replacement name of *Bruandia* Tutt and then Roh and Byun (2017) assigned *niphonica* in *Bruandella*. Saigusa and Sugimoto (2013) discussed differences between *niphonica* Hori collected from the type locality and *Psychecasta* (Pallas) in detail, pointing out that the intercalary cell in the male forewing is present in *niphonica* Hori, whereas absent in *Psychecasta* (Pallas). Recently, Saigusa and Sugimoto (2022) desginated the lectotype specimen (*Fumeaniphonica* Hori, 1926) and concluded that it was reasonable for this species to be a new combination into the genus *Bruandella*. Therefore, *niphonica* Hori is a valid species. We accepted this point of view. Furthermore, the molecular analyses (Table 2, Figs. 2 and 3) supported specimens from Korea and Japan representing a valid species in *Proutia*, treated as *Proutianiphonica* (Hori, 1926), comb. nov.

## Checklists

### A checklist of the genus *Proutia* in Korea

#### Proutia maculatella

Saigusa and Sugimoto, 2014

11F9BC24-D04B-58D3-8DF7-BCCD63101280

Proutia maculatella Saigusa and Sugimoto, 2014: 144; Roh et al. (2016): 673. Type locality: Japan.

##### Distribution

Korea, Japan.

##### Notes

This species was first reported by Roh et al. (2016) in Korea.

#### Proutia nigra

Saigusa and Sugimoto, 2014

F39E418C-9DA8-5A6B-ADBC-B92B115CB501

Proutia nigra Saigusa and Sugimoto, 2014: 149; Roh and Byun (2017): 226. Type locality: Japan.

##### Distribution

Korea, Japan.

##### Notes

This species was first reported by Roh and Byun (2017) in Korea.

#### Proutia niphonica

(Hori, 1926), comb. nov.

4BBF1B59-06A3-5D6E-AA8A-815E0755BABD

Eumea niphonica Hori, 1926: 28 (*Eumea* is misspelling of *Fumea*). Type locality: Japan.
Psyche casta (Pallas, 1767): Sauter and Hättenschwiler (1991): 79; Leraut (1997): 87; Sobczyk (2011): 257.
Bruandia niphonica (Hori, 1926): Sugimoto (2009a): 12; Saigusa and Sugimoto (2013): 145.
Bruandella niphonica (Hori, 1926): Saigusa and Sugimoto (2014): 143; Roh and Byun (2017): 224; Saigusa and Sugimoto (2022): 147.

##### Distribution

Korea, Japan.

##### Notes

This species was first reported by Roh and Byun (2017) in Korea.

#### Proutia cornucervae

Roh & Lee, sp. nov.

80D1AEE7-768F-55C0-81DF-2B045804069E

##### Distribution

Korea (new species).

## Analysis

A total of 22 new sequences was generated from four species of *Proutia* and one species of *Psyche* (567–658 bp of partial *COI*). All new sequences were deposited in GenBank (accession numbers: MT154331–154332, OR122630–122634 and OR134240–134253 in Table 1). The DNA barcodes (*COI*) were compared to those of 50 specimens in 13 species, 25 sequences downloaded from NCBI and three sequences downloaded from BOLD systems.

Genetic divergence of *COI*, using uncorrected p-distances amongst the *Proutia* and *Psyche* species, ranged from 5.8% to 16.0% (the result between *P.betulina* and *P.rotunda* was excluded, as it was considered to be due to misidentification), while intraspecific divergence ranged from 0% to 3.6% (Table 2). The molecular analyses (p-distance, NJ and PA analyses) revealed that *Proutia* sp. and *P.maculatella* were closely related to *P.cornucervae* sp. nov. (Table 2, Figs 2, 3). The maximum difference amongst populations within *Proutia* sp. was 1.3% and within *P.maculatella* 1.0% (Table 2). Genetic divergence between *P.cornucervae* sp. nov. and its molecularly related species *Proutia* sp. and *P.maculatella* are 6.2% and 6.5%, respectively and that strongly supported the separation of *P.cornucervae* sp. nov. and its congeners (Table 2).

## Supplementary Material

XML Treatment for Proutia cornucervae

XML Treatment for Proutia niphonica

XML Treatment for Proutia maculatella

XML Treatment for Proutia nigra

XML Treatment for Proutia niphonica

XML Treatment for Proutia cornucervae

## Figures and Tables

**Table 1.** Species with DNA barcodes (*COI*) and GenBank and BOLD systems accession numbers used in this study.

Species	Country	Accession No.	Species	Country	Accession No.
*Proutiamaculatella*	Korea	OR122630 ^*^	*P.comitella*	Slovenia	KX045500
”	Korea	OR122631 ^*^	”	Italy	KU497314
”	Korea	OR122632 ^*^	”	Italy	KU497313
”	Korea	OR122633 ^*^	*Proutianiphonica*	Korea	MT154326 ^*^
”	Korea	OR122634 ^*^	”	Korea	OR134246 ^*^
”	Korea	OR134240 ^*^	”	Korea	OR134247 ^*^
”	Korea	OR134241 ^*^	”	Korea	OR134248 ^*^
”	Japan	LC094176	”	Korea	OR134249 ^*^
*P.cornucervae* sp. nov.	Korea	MT154331 ^*^	”	Korea	OR134250 ^*^
*P.nigra*	Korea	OR134242 ^*^	”	Korea	OR134251 ^*^
”	Korea	OR134243 ^*^	”	Korea	OR134252 ^*^
”	Korea	OR134244 ^*^	”	Korea	OR134253 ^*^
”	Korea	OR134245 ^*^	”	Japan	LC094173
*P.norvegica*	Norway	BOLD:AAD0603	”	Japan	LC094187
”	Norway	BOLD:AAD0603	*Proutia* sp.	Japan	LC094174
*P.raiblensis*	Austria	BOLD:AAQ1159	”	Japan	LC094178
”	Slovenia	KX044945	*Psychecasta*	Germany	HQ563536
	Slovenia	KX047374	”	Austria	KM572052
*P.betulina*	Slovenia	KX046545	”	Canada	GU096047
	Austria	KP253124	”	Finland	HM873163
	Germany	HQ563535	*P.yeongwolensis*	Korea	MT154332 ^*^
	Slovenia	KX045856	*P.crassiorella*	Austria	KM572313
*P.rotunda*	Finland	JF853593	”	Italy	HM914070
	Denmark	KX043914	”	Finland	HM875551
*P.comitella*	Italy	KX045446	”	Denmark	KX043231

* In this study

**Table 2.** Inter-and intraspecific genetic differences in the two genera *Proutia* and *Psyche* species for *COI* (658 bp), calculated using *p*-distances.

	**1**	**2**	**3**	**4**	**5**	**6**	**7**	**8**	**9**	**10**	**11**	**12**	**13**
1	0- 0.02												
2	0.093- 0.103	0											
3	0.124- 0.134	0.119- 0.121	0.003- 0.009										
4	0.110- 0.120	0.113- 0.116	0.058- 0.060	0									
5	0.105- 0.117	0.109- 0.119	0.102- 0.109	0.099- 0.105	0.002- 0.006								
6	0.105- 0.115	0.109- 0.114	0.103- 0.105	0.099- 0.100	0- 0.005	0							
7	0.112- 0.128	0.117- 0.121	0.099- 0.107	0.096- 0.099	0.093- 0.102	0.093- 0.097	0.002- 0.010						
8	0.113- 0.123	0.121- 0.126	0.106- 0.108	0.092- 0.099	0.091- 0.096	0.091- 0.094	0.059- 0.070	0.013					
9	0.116- 0.123	0.122- 0.124	0.102- 0.103	0.106- 0.108	0.103- 0.108	0.103	0.062- 0.068	0.065- 0.072	0				
10	0.119- 0.131	0.120- 0.127	0.108- 0.118	0.102- 0.108	0.114- 0.129	0.114- 0.125	0.067- 0.079	0.082- 0.093	0.091- 0.102	0- 0.019			
11	0.128- 0.143	0.138- 0.141	0.146- 0.147	0.137- 0.141	0.123- 0.128	0.123	0.125- 0.134	0.134- 0.140	0.131- 0.132	0.135- 0.144	0- 0.018		
12	0.137- 0.149	0.135- 0.141	0.129- 0.141	0.132- 0.138	0.125- 0.141	0.125- 0.137	0.131- 0.141	0.134- 0.147	0.137- 0.138	0.144- 0.155	0.075- 0.085	0- 0.036	
13	0.147- 0.152	0.148- 0.150	0.150- 0.153	0.139- 0.140	0.132- 0.137	0.132	0.138- 0.144	0.146- 0.149	0.160	0.146- 0.157	0.120	0.128- 0.132	0

1, *Proutianiphonica*; 2, *Proutiacomitella*; 3, *P.raiblensis*; 4, *P.norvegica*; 5, *P.betulina*; 6, *P.rotunda*; 7, *P.maculatella*; 8, *Proutia* sp.; 9, *P.cornucervae* sp. nov.; 10, *P.nigra*; 11, *Psychecasta*; 12, *P.crassiorella*; 13, *P.yeongwolensis*.
